# Intestinal Microbiota as a Contributor to Chronic Inflammation and Its Potential Modifications

**DOI:** 10.3390/nu13113839

**Published:** 2021-10-28

**Authors:** Marta Potrykus, Sylwia Czaja-Stolc, Marta Stankiewicz, Łukasz Kaska, Sylwia Małgorzewicz

**Affiliations:** 1 Department of General, Endocrine, and Transplant Surgery, Faculty of Medicine, Medical University of Gdansk, Smoluchowskiego 17, 80-214 Gdansk, Poland; lukasz.kaska@gumed.edu.pl; 2 Department of Clinical Nutrition, Faculty of Health Sciences, Medical University of Gdansk, Dębinki 7, 80-211 Gdansk, Poland; sylwia.czaja-stolc@gumed.edu.pl (S.C.-S.); marta.stankiewicz@gumed.edu.pl (M.S.); sywia.malgorzewicz@gumed.edu.pl (S.M.)

**Keywords:** microbiome, inflammation, intestinal epithelial barrier, diet, prebiotic, probiotic

## Abstract

The gut microbiota is a crucial factor in maintaining homeostasis. The presence of commensal microorganisms leads to the stimulation of the immune system and its maturation. In turn, dysbiosis with an impaired intestinal barrier leads to accelerated contact of microbiota with the host’s immune cells. Microbial structural parts, i.e., pathogen-associated molecular patterns (PAMPs), such as flagellin (FLG), peptidoglycan (PGN), lipoteichoic acid (LTA), and lipopolysaccharide (LPS), induce inflammation via activation of pattern recognition receptors. Microbial metabolites can also develop chronic low-grade inflammation, which is the cause of many metabolic diseases. This article aims to systematize information on the influence of microbiota on chronic inflammation and the benefits of microbiota modification through dietary changes, prebiotics, and probiotic intake. Scientific research indicates that the modification of the microbiota in various disease states can reduce inflammation and improve the metabolic profile. However, since there is no pattern for a healthy microbiota, there is no optimal way to modify it. The methods of influencing microbiota should be adapted to the type of dysbiosis. Although there are studies on the microbiota and its effects on inflammation, this subject is still relatively unknown, and more research is needed in this area.

## 1. Introduction

Berg’s publication was intended to systematize the definition of the microbiome. Based on many definitions, the authors proposed an extended one, in which the microbiome is presented as a combination of microbiota and their theater of activity. Microbiota are living organisms and include bacteria, archaea, fungi, protists, and algae. Viruses, phages, plasmids, viroids, prions, and free DNA or RNA are, by definition, not living organisms, so they are not members of the microbiota, but they belong to the microbiome. The term theater of activity refers to the structural elements of microorganisms, their metabolites, and molecules produced by the host and modified by environmental conditions. The microbiome also includes the given environmental conditions. All of these factors create a complex micro-ecosystem on which the health and well-being of the host largely depend [1].

The gastrointestinal tract is inhabited by more than 10^14^ microorganisms, most of which have not been identified [2]. The bacteria are classified into 12 different types, of which 93.5% belong to 4 types: *Firmicutes*, *Bacteroidetes*, *Proteobacteria*, and *Actinobacteria* [3]. The gut microbiota plays a major role in digestion, regulation of the immune system, and production of compounds that might alter human metabolism. The microbiota also performs many other functions, i.e., synthesizing vitamins, creating appropriate environmental conditions influencing the oxygen level and pH in the intestines, competing with pathogens (thus reducing their number), and stabilizing the intestinal barrier [4].

The scientific literature shows that the intestinal microbiota can stimulate the immune system and, in the case of excessive stimuli, affect the formation of inflammation. Therefore, the current study aimed at overviewing and summarizing the literature concerning the association between microbiota and chronic inflammation. The main purpose of the current study is to locate articles on a given topic and organize knowledge based on already existing publications. It was necessary to review published evidence to establish what is known about intestinal microbiota status, the mechanism of developing chronic inflammation, and the benefits of dietary modifications and pre/probiotic supplementation. For this purpose, the Pubmed and World Wide Science databases were searched. While searching the databases, the keywords “microbiota”, “intestinal barrier”, “inflammation”, “probiotics”, “prebiotics”, and “diets” were used, and by combining them into various combinations, it was possible to search for articles that fit the topic of the manuscript. In addition, articles included in the bibliography of the selected publications were also searched. The timeframe for all publications was established from 2000 until 2021. The exclusion criteria were articles published earlier than 2000 and articles written in a language other than Polish or English.

## 2. Intestinal Barrier

The intestinal barrier is necessary to separate the external environment in the intestines from the interior of the host’s body. The gastrointestinal epithelium is the largest surface that comes into direct contact with the external environment, which causes it to be highly vulnerable to harmful factors. The intestinal barrier allows selective permeability; it prevents the passage of antigens from the intestine into the bloodstream and allows the absorption of water and nutrients [5]. Apart from a single layer of epithelial cells interspersed with intraepithelial lymphocytes, the intestinal barrier consists of a layer of mucus and the underlying mucosal immune system. The cells of the immune system are abundantly distributed in the lamina propria, which makes it an important link between the microbiome and the immune system [6].

The intestinal epithelial cells are not homogeneous; they consist of enterocytes, goblet cells (GCs), Paneth cells (PCs), tuft cells, microfold (M) cells, and enteroendocrine cells, each of which has different specialized functions [7]. Enterocytes are connected via tight junctions (TJs), adherens junctions (AJs), gap junctions, and desmosomes, whose role is to secure selective intestinal permeability and to regulate intracellular interactions among epithelial cells [7]. Impaired functioning of intercellular junctions leads to increased intestinal permeability, resulting in increased transport of inflammatory mediators, which may contribute to chronic intestinal inflammation. Tight junctions are mainly responsible for the permeability of the intestinal epithelium. They are composed of occludins, claudins, adhesive proteins, tricellulins, and zonula occludens proteins [8,9]. Inflammation is a factor that exacerbates TJ disorders, which leads to reduced gut tightness [7].

Goblet cells secrete mucin, providing epithelial cells with a mucus lining, which creates a physical barrier from microorganisms living in the lumen. Different types of mucins are produced depending on the location of the goblet cell; in the intestines, mucin 2 (MUC2) is mainly secreted. Another factor produced by GCs that protects endothelial cells against contact with microbes is the resistin-like molecule β (RELM-β). RELM-β reduces the number of microorganisms in the mucus layer through bactericidal abilities and limits the development of intestinal parasites. The goblet cells also secrete zymogen granule protein 16 (ZG16), which aggregates bacteria and prevents their adherence to the epithelium [10].

Other highly specialized endothelial cells are the Paneth cells, which are predominantly located in the crypts of Lieberkun. PCs produce some substances with various antimicrobial properties. Lysozyme acts against Gram-positive bacteria and activates the innate and the acquired system. Defensins have a wider range of action and may eliminate not only Gram-positive bacteria but also Gram-negative bacteria, as well as fungi and viruses. The role of C-type antibacterial lectins, regenerating gene family protein III β and γ (RegIIIβ, RegIIIγ), is to prevent Gram-positive bacteria from attaching to the mucosa. Other antimicrobial molecules produced by PCs are angiogenin-4, secretory phospholipase A2 (sPLA2), α1-antitrypsin, CRP-duktin, and RELM-β. The secretion of the above-mentioned compounds depends on the presence of microorganisms and, more precisely, on their number and type. PCs can recognize microbial molecules due to the presence of different types of receptor pattern recognition receptors (PRRs): nucleotide-binding and oligomerization domain (NOD)-like proteins and toll-like receptors (TLRs). Activation of NOD-like receptor (NLR) contributes to the secretion of lysozyme and defensins, while the activation of TLR releases other antimicrobial compounds [11].

Membranous or M cells overlay organized lymphoid follicles spread over the entire length of the intestine. They transport microorganisms and other antigens to the lymphoid tissue, playing a key role in the initiation of the immune response [12]. The contact of antigen with dendritic cells, underlying the M cell, contributes to the production of antigen-specific immunoglobulin A (IgA). IgA restricts potentially harmful antigens from entering the gut epithelia [13]. Tuft cells have been proven to be important in the host’s response to exposure to common eukaryotes, such as protists and helminths. A parasitic infection triggers the secretion of interleukins, particularly interleukin 25 (IL-25), which, in turn, causes the secretion of interleukin 13 (IL-13), which is responsible for goblet cell hyperplasia and mucin production [14].

Enteroendocrine cells (EECs) can be divided into more than 10 types of cells based on which hormones they secrete. EECs, through the production of hormones, take part in the processes regulating the absorption of nutrients, the tightness of the intestinal barrier, the response of the immune system, visceral hyperalgesia, and intestinal motility. EECs express G protein-coupled receptors (GPCRs), whose ligands are microbiota-derived short-chain fatty acids (SCFAs). By binding with the GPCRs, butyrate contributes to the secretion of glucagon-like peptide 2 (GLP-2), which directly affects the intestinal endothelial cells and causes their proliferation [15].

Another component that protects the body against the excessive development of pathogens is the intestinal lumen environment. The lumen of the digestive tract is heterogeneous, and its conditions depend on the functions and localization of individual segments. The pH values in the stomach fluctuate between pH 1.4 and 4.6. The acidity of the environment depends on its distance from the stomach and the secretion of alkaline pancreatic and liver juices. The antibacterial properties of gastric juice are based on the content of hydrochloric acid as well as other compounds, e.g., enzymes, such as gastric trypsin. Bile acids released in the proximal part of the duodenum are metabolized in the colon by microorganisms to secondary bile acids, which have antibacterial properties and may affect intestinal permeability. Other factors limiting the excessive growth of microorganisms are the oxygen content and the availability of nutrients [16].

The gut microbiota is also a significant component of the gut barrier. On the one hand, commensal microbes rival pathogenic microorganisms, competing with them for a place of settlement and nutrients. On the other hand, they induce epithelial cells to proliferate. Activation of TLRs is necessary for increased proliferation following intestinal injury. Additionally, toll-like receptor 2 (TLR2) signaling contributes to enhancement of TJs in the intestinal epithelium. Furthermore, activation of NOD-1 by peptidoglycan initiates the formation of isolated lymphoid follicles in the gut [17]. The synthesis of antimicrobial molecules in Paneths cells is also dependent on the TLR/myeloid differentiation factor 88 (MyD88) pathway [18]. The mere presence of microorganisms in the intestines initiates mechanisms that limit the excessive development of microbiota. However, microbiota influences the state of the intestinal barrier also indirectly through its metabolites. A crucial metabolic activity of the microbiota is the production of SCFAs by fermenting carbohydrates that are not digestible by humans. SCFAs activate G protein-coupled receptors 41 (GPR41) and 43 (GPRs43), which affect the expression of TJ proteins and regulation of endocrine cells. Butyrate nourishes the intestinal epithelial cells and increases the production of mucin, which improves the function of the intestinal barrier [3]. Epithelial cells and cells lying above the mucus layer limit the contact of substances in the intestinal lumen with the immune system but do not prevent it completely. The presence of microbiota in the gut is essential for the proper maturation of the immune system. A moderate amount of bacterial endotoxins contributes to the maturation of regulatory T cell lymphocytes and facilitates the neutralization of pathogenic microbes. However, increased translocation of microorganisms leads to inflammation [19].

## 3. Microbiota-Derived Inflammation

Inflammation is a natural response of the body that is triggered by harmful stimuli, and its evolutionary purpose is to restore homeostasis. Inflammation can be triggered by external and internal inducers. The external inducers are divided into microbial or non-microbial factors. Non-microbial factors include allergens, irritants, and toxic compounds. Two main microbial factors that trigger inflammation are pathogen-associated molecular patterns (PAMPs) and virulence factors. Virulence factors are molecules that occur in pathogens [20]. PAMPs, however, are small molecules with conserved patterns that occur among various microorganisms. PAMPs include components that build cell walls, including bacterial peptidoglycan, lipopolysaccharide (LPS), lipoteichoic acid (LTA), and flagellin [21]. Other factors classified as PAMPs include intracellular pathogens like viral RNA or DNA. PAMPs are recognized by pattern recognition receptors (PRRs), which occur on the surface and in the cytosol of immune and epithelial cells [22]. The families of receptors belonging to PRRs are TLR, NLR, C-type lectin receptor (CLR), retinoic acid-inducible gene (RIG)-I-like receptor (RLR), and the absent in melanoma 2 (AIM2)-like receptor (ALR). The bond of PAMPs with their respective specific receptors activates the innate immune system, which plays a crucial role in first-line defense [23].

### 3.1. Pathogen-Associated Molecular Patterns

**Figure 1:**
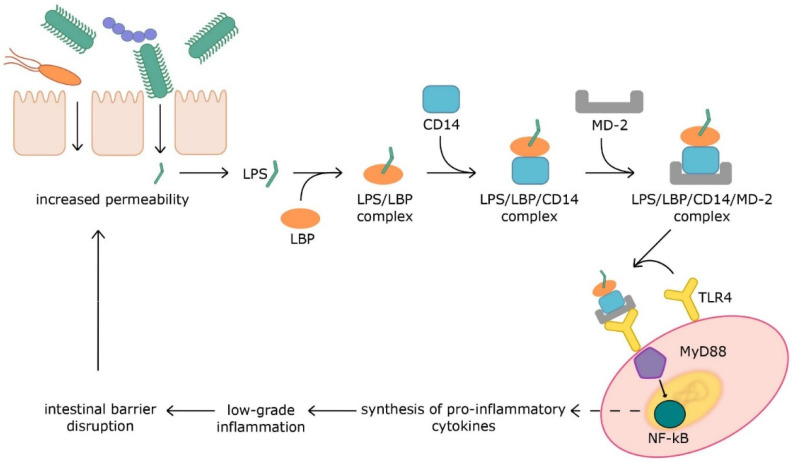
Mechanism of LPS’ influence on inflammation and intestinal permeability. Aberrations: LPS—lipopolysaccharide; LBP—LPS-binding protein; CD14—cluster of differentiation 14; MD-2—myeloid differential factor 2; TLR4—toll-like receptor 4; MyD88—myeloid differentiation factor 88; NF-κB—nuclear factor kappa-light-chain-enhancer of activated B cells.

LPS is a component of the outer cell wall of Gram-negative bacteria and is the best-known bacterial endotoxin. It protects bacteria against harmful factors, such as antibiotics or immune cells, of the host from the external environment. The membrane receptor CD14 is on the surface of cells of the immune system and epithelial cells. It is also available in a soluble form in serum. Lipopolysaccharide binds to a lipopolysaccharide-binding protein (LBP) to increase the affinity for CD14 receptors. The LPS/LBP/CD14 complex, in turn, binds to the myeloid differentiation factor 2 (MD-2) and, in this form, is recognized by the toll-like receptor 4 (TLR4). Activation of this receptor leads to the release of mediators including MyD88, which stimulates nuclear factor κB (NF-κB) to produce proinflammatory cytokines, including tumor necrosis factor α (TNF-α) and interleukin 1β (IL-1β) [19]. Paradoxically, inflammation enhances intestinal barrier disruption and causes increased gut permeability, which exacerbates chronic low-grade inflammation [24]. This process is shown in a simplified way in Figure 1. The increased plasma LPS levels associated with chronic inflammation are called metabolic endotoxemia. This condition leads to the development of cardiometabolic diseases. Endotoxemia is characterized by 2–3-fold higher serum LPS levels (10–15-fold lower serum LPS levels than in sepsis), which is a life-threatening condition [25]. Endotoxemia is associated with a variety of diseases, including obesity, type 2 diabetes, non-alcoholic fatty liver disease, chronic kidney disease, and cardiovascular disease [26].

The receptor that recognizes the bacterial protein of flagella (flagellin) is toll-like receptor 5 (TLR5). Flagellum, a microbial component for locomotion, is mainly found in Gram-negative bacteria [27]. TLR5 is produced in immune cells and epithelial cells of the gastrointestinal tract, respiratory tract, and liver. It is mainly expressed on the basolateral side of gut epithelial cells, and its role is to detect the translocation of bacteria across the endothelial barrier [28]. The activation of TLR5 by flagellin results in the induction of the synthesis of chemokines, nitric oxide (NO), hydrogen peroxide (H_2_O_2_), and proinflammatory cytokines [27]. Overactivation of TLR5 may contribute to impairment of the intestinal barrier integrity and cause chronic inflammation [28]. The composition of the microbiota associated with inflammation is characterized by an increased number of motile bacteria, including bacteria with flagella, which more easily penetrate the mucosa and initiate an inflammatory response. A study using a mouse model showed that administration of anti-flagellin antibodies prevents interleukin 10 (IL-10) deficiency-induced colitis and reduces diet-induced obesity [29]. Lodes et al. found that bacterial flagellin was a dominant antigen in mice with Crohn’s disease, a disease characterized by chronic intestinal inflammation [30].

Peptidoglycan (PGN) is a significant cellular structure that protects bacteria from environmental factors. It is also necessary for bacterial growth. Peptidoglycan is a complex polymer consisting of a network of glycan strands that gives the cell mechanical strength. PGN also consists of other compounds, such as lipoproteins, polysaccharides, and glycolipids, that give bacteria various physical and chemical properties. The Gram-negative and Gram-positive bacteria have a differently organized peptidoglycan structure. In the first group, peptidoglycan is thinner and is located between the outer and cytoplasmic membranes. However, in Gram-positive bacteria, peptidoglycan is significantly thicker and occurs on the outer side of the cytoplasmic membrane, creating the outermost barrier separating the cell from the environment [31]. Mammals produce four peptidoglycan recognition proteins (PGLYRPs), which are secreted by immune and epithelial cells and lead to bacterial cell wall lysis [32]. The peptidoglycans found in the intestinal lumen activate Paneth cells to synthesize defensins, which allows the regulation of the microbiota and protection against pathogens, such as *Salmonella enterica*. The recognition of peptidoglycan by NOD1 and NOD2 receptors leads to activation of NF-κB and the mitogen-activated protein kinase (MAPK). Both pathways contribute to the transcription of proinflammatory genes, leading to the synthesis of cytokines, adhesion molecules, and inflammatory mediator enzymes. Another signaling pathway involving the activation of cryopyrin, an NOD family protein, also leads to the activation of NF-κB as well as caspase-1-dependent maturation and secretion of the cytokines IL-1β and interleukin 18 (IL-18) [33]. In 2019, the journal *Nature* published an article in which researchers inhibited the development of autoimmune encephalomyelitis and autoimmune arthritis in mice by neutralizing peptidoglycans in the circulatory system. This suggests that peptidoglycans may participate in the pathogenesis of autoimmune diseases [34].

**Figure 2:**
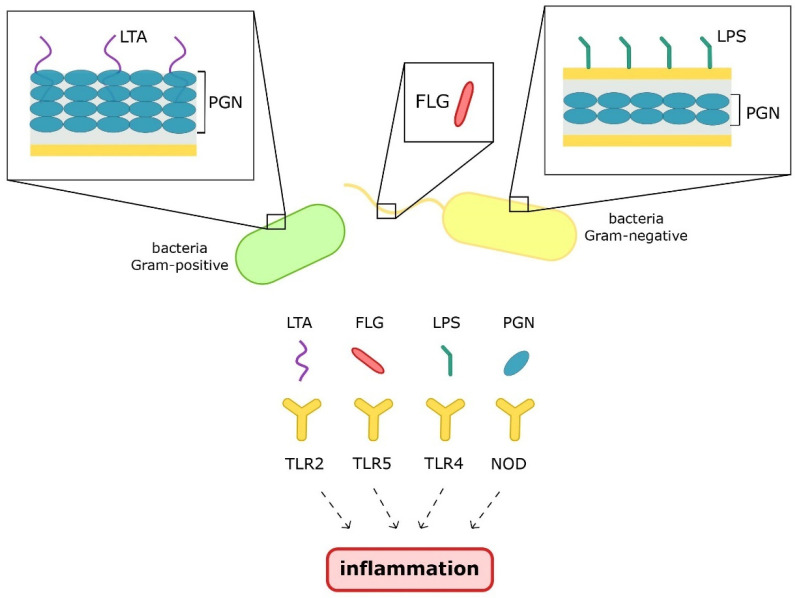
Origin and receptors of pathogen-associated molecular patterns. Aberrations: LTA—lipoteichoic acid; PGN—peptidoglykan; FLG—flagellin; LPS—lipopolisaccharide; TLR2/4/5 –toll-like receptors2/4/5; NOD—nucleotide-binding oligomerization domain.

LTA, a component of a Gram-positive bacteria’s cell wall, is recognized by TRL2 and leads to immune system activation and the development of adaptive immunity. Similar to the other PAMPs, in the case of an excessive immune response, inflammation also develops [35]. LTA is recognized by TLR-2 and, thus, activates the signaling pathway, leading to the expression of cyclooxygenase, which contributes to prostaglandin E2 (PGE2) synthesis [36]. PGE2, via activation of mast cells, elicits edema formation and vascular permeability, two main symptoms of inflammation. It is also involved in gene regulation leading to cytokine signaling [37]. In macrophages, LTA has been established to stimulate the release of interleukin 1 (IL-1), interleukin 6 (IL-6), and TNF-α [36]. The origin of PAMPs and their recognizing receptors are presented graphically in Figure 2.

### 3.2. Metabolites

Microorganisms can interfere with the host’s immune system and contribute to inflammation not only through their structural elements but also through the products of their metabolism. Short-chain fatty acids are related to several processes that positively influence the metabolism of the host. Butyrate nourishes intestinal endothelial cells and increases the thickness of mucin, which improves the tightness of the intestinal barrier [4]. Propionate stimulates L-enteroendocrine cells to release glucagon-like peptide 1 (GLP-1) and peptide tyrosine-tyrosine (PYY), which leads to inhibition of appetite. In the liver, it inhibits the synthesis of cholesterol and fatty acids, which reduces the chances of developing obesity and associated diseases. However, not all SCFAs have a beneficial effect on the host. Acetate is attributed to properties that worsen the metabolic state and contribute to the formation of obesity. In the liver, acetate contributes to the synthesis of lipids, which can lead to dyslipidemia. This compound also increases the appetite by increasing the production of gastric ghrelin [38].

Trimethylamine N-oxide (TMAO), a bacterial metabolite produced during choline, betaine, and L-carnitine refinement, is associated with an increased risk of cardiovascular disease. It has been established that elevated levels of TMAO induce NLR family pyrin domain-containing 3 (NLRP3) activation. NLRP3 belongs to the inflammasomes, which are intracellular protein complexes responsible for initiating inflammatory processes. Their function is to regulate the maturation and secretion of the proinflammatory cytokines IL-1β and IL-18 through the activation of caspase-1. In addition, TMAO, by activating NF-κB, causes the synthesis of proinflammatory proteins with pro-atherosclerotic properties, such as cyclooxygenase 2 (COX2), E-selectin, IL-6, and intracellular adhesion molecule 1 [39].

The intestinal microbiota cause the deconjugation of bile acids in the intestine, resulting in the formation of secondary bile acids, including deoxycholic acid (DCA). It has been shown that a high-fat diet can increase DCA levels up to 10 times. Increased DCA levels are associated with impaired intestinal epithelial integrity along with gut inflammation. It has been indicated that the development of intestinal inflammation is due to the activation of the NLRP3 inflammasome [40]. In addition, high levels of secondary bile acids (BAs) contribute to the formation of reactive oxygen and nitrogen species. BAs also damage cell membranes, mitochondria, and DNA, which may result in an increased risk of colon cancer. Bacterial endotoxins and metabolites can synergistically increase their harmful properties. The accumulation of DCA and LTA amplifies signals caused by TLR2 activation, leading to overproduction of COX2, which is associated with the suppression of natural killer T and dendritic cells and may lead to cancer and inflammatory diseases [41].

The preferred energy source for most gut microbiota is carbohydrates, but if carbohydrates are insufficient in the colon, proteins are fermented. The products of these transformations are numerous toxic bacterial metabolites, including p-cresol sulfate (pCS) and indoxyl sulfate (IS) [42]. An increase in IS and pCS levels in serum is characteristic of patients with chronic kidney disease. In these patients, a positive correlation has been found between circulating IS and pCS and vascular stiffness and calcification, which means a greater risk of cardiovascular disease and the progression of chronic kidney disease. In turn, TMAO, IS, and pCS (as compounds with uremic toxicity) lead to dysbiosis and increased intestinal permeability, which intensifies the inflammation that is already taking place in the host organism [43].

## 4. Modifications of Microbiota and Its Impact on the Inflammatory Profile

Dysbiosis and the accompanying increased intestinal permeability lead to the development of low-grade chronic inflammation, which is a key contributor to metabolic disorders and obesity. Therefore, modification of the microbiota may reduce inflammation and improve metabolic status.

### 4.1. Diets and Nutrients

#### 4.1.1. Mediterranean Diet

The Mediterranean diet (MD) is considered a healthy eating style that reduces the risk of cardiovascular and metabolic diseases and cancer. The diet model includes the consumption of whole grains, legumes, fresh vegetables and fruits, olive oil, nuts, seeds, a moderate amount of fish, and a small amount of dairy products and meat [44]. The MD’s pro-health nature consists of lipid-lowering, anti-inflammatory, antioxidant, and anticancer properties. Recent studies indicate that the MD also influences metabolism by influencing the gut microbiota [45,46]. MD modulates the composition of the gut microbiota and reduces endotoxemia [47]. In a study by Haro et al., it was observed that adherence to the MD for two years by obese men with metabolic syndrome increased the number of bacteria of the genus *Bacteroides* and *Prevotella*, and saccharolytic bacteria of the genus *Roseburia*, *Ruminococcus*, *Parabacteroides distasonis*, and *Faecalibacterium prausnitzii* [48]. An 8-week dietary intervention based on MD in overweight and obese people led to an increase in the number of *Faecalibacterium prausnitzii* and reduction of *Ruthenibacterium lactatiformans*, *Flavonifractor plautii*, *Parabacteroides merdae*, *Ruminococcus torques*, and *Ruminococcus gnavus*. Following the Mediterranean diet was associated with an increased concentration of SCFA in the feces. Significantly lower levels of high-sensitivity C-reactive protein (hsCRP) were reported in people with a greater variety of bacterial genomes [49]. Tagliamonte et al. compared the effects of the Mediterranean and Western diets on the gut microbiome. After eight weeks, there was a significant increase in the amount of *Roseburia faecis* and *R. hominis* in the MD compared to the Western diet. Moreover, the amount of *Akkermansia muciniphila* increased in the MD group. The MD lowered plasma arachidonoylethanolamide (AEA). In this mechanism, MD may show anti-inflammatory effects by increasing the tightness of the intestinal barrier [50]. Adherence to the Mediterranean diet was negatively correlated with serum lipopolysaccharide concentration in patients with atrial fibrillation. The single nutrients correlated with decreased endotoxemia were fruit and legumes [51]. The factors responsible for the effect of MD on the intestinal microbiota are dietary fiber, the advantage of plant vs. animal proteins, unsaturated fatty acids, and polyphenols [47].

#### 4.1.2. Vegetarian/Vegan Diets

Several studies have proved that vegetarian and vegan diets have a positive effect on human health. The elimination of meat or all animal products reduces the risk of developing cardiovascular diseases, diabetes, cancer, and metabolic syndrome. Plant-based diets also affect the composition of the intestinal microbiota; however, the research results are inconclusive. In people on a plant-based diet, the amounts of *Bifidobacteria*, *Escherichia coli*, and *Enterobacteria* were lower than in omnivores [52,53]. It was observed that the ratio of *Prevotella*-to-*Bacteroides* (P/B) was lower in those who consumed more fiber and starch than in those following the Western diet [54,55,56]. Plant-based diets increased the number of *Bacteroidetes* and reduced *Firmicutes*. This ratio is beneficial in the prevention and treatment of obesity. In addition, increases in the levels of *Faecalibacterium prausnitzii* and *Clostridium clostridioforme* were noted [57]. In a study by Trefflich et al., SCFA concentrations did not differ significantly between vegans and omnivores. Fecal pH and ammonia levels were lower in vegans compared to omnivores [58]. Vegans had a higher number of *Roseburia* and *Faecalibacterium*, which produce butyrate, the main source of energy for the colonocytes, which may result in improved intestinal barrier integrity. This shift in microbiota was associated with lower serum levels of LPS and parameters of inflammation (CRP, TNF-α) in vegans compared to omnivores [59,60].

#### 4.1.3. Gluten-Free Diet

In recent years, the use of a gluten-free diet (GFD) has become very popular, but the medical indications for its use are only celiac disease (CD) and non-celiac gluten sensitivity. Intestinal dysbiosis develops in people with CD who do not use GFD. The levels of pathogenic Gram-negative bacteria, such as *Klebsiella*, *Prevotella*, and *Serratia*, are increased and the levels of *Bifidocteria* and *Firmicutes* are lower than in healthy people [61,62]. CD patients using GFD have lower species diversity of bacteria and less variety of *Lactobacillus* and *Bifidobacterium* species. However, the concentration of SCFA in this group is similar to those in healthy people [63,64]. Differences in the composition of the gut microbiota were observed in people with and without CD-related gastrointestinal symptoms despite GFD use. In the group with gastrointestinal symptoms, the amount of *Prevotella* was higher along with the lower number of *Bacteroidetes* and *Firmicutes* compared to microbiota in people without symptoms [65]. The use of GFD by healthy people reduced the amounts of *Lactobacillus*, *Bifidobacterium*, and *Faecalibacterium prausnitzii*, and increased the amounts of *Escherichia coli* and *Enterobacteriaceae* [66,67]. The cause of these disorders is not yet known, but it has been hypothesized that gluten has prebiotic properties, and its elimination impairs the growth of health-promoting bacteria [68].

#### 4.1.4. Fiber

Many of the health-promoting properties of plant-based diets are due to the higher content of dietary fiber compared to the Western diet. Fiber is mainly cellulose, pectins, dextrins, waxes, and lignans. Some fractions of fiber are classified as prebiotics [69]. Fiber is mainly found in whole grains, legumes, fruits, and vegetables. Fiber is not digested in the digestive tract. In the large intestine, it is fermented by intestinal bacteria. It stimulates the growth of many types of bacteria and is the main substrate for the synthesis of postbiotics, such as SCFA [70]. In a systematic review and meta-analysis by So et al., a significant increase in the number of *Bifidobacterium* spp. was observed on the basis of 59 studies involving nearly 1900 people. An increase in the number of *Lactobacillus* spp. was noticed on the basis of 28 studies involving approximately 850 people [71]. In a study carried out among the elderly, it was demonstrated that in the group consuming a diet with a higher fiber content, the diversity of microbiota was significantly higher compared to people whose diets were high in fat and low in fiber. Inflammatory parameters, such as CRP, IL-6, and TNF-α, were significantly higher in the group consuming the low-fiber diet [72].

### 4.2. Prebiotics

A prebiotic is defined as ‘a substrate that is selectively utilized by host microorganisms conferring a health benefit’. Prebiotics must meet the following criteria: they must be resistant to gastric pH, they cannot be hydrolyzed by mammalian enzymes and absorbed in the gastrointestinal tract, they can be fermented by gut microbiota, and they must selectively stimulate the growth of intestinal bacteria. Prebiotics decrease the intestinal pH and maintain water’s retention in the intestine [73]. The main group of prebiotics is carbohydrates, including oligosaccharides and polysaccharides. The most popular of them are fructooligosaccharides (FOS) and galactooligosaccharides (GOS). Prebiotics also include polyols, phenolic compounds, unsaturated fatty acids-conjugated linoleic acid (CLA), and polyunsaturated fatty acids (PUFAs). Fiber is considered a good source of prebiotics, but only some of its compounds meet the criteria of a prebiotic. A term for carbohydrates that are the main source of energy for the intestinal microbiota is microbiota-accessible carbohydrates (MACs) [74].

#### 4.2.1. Fructooligosaccharides

FOS are short-chain fructans constructed of 2-10 fructofuranose residues, which are connected by β bonds. Natural sources of FOS include artichokes, onions, asparagus, wheat, bananas, potatoes, and honey [75]. FOS supplementation mainly stimulates the growth of *Bifidobacteria* sp. and *Lactobacillus* sp. [76,77]. Supplementation of FOS in patients with Crohn’s disease led to a significant increase in the concentration of fecal bifidobacteria [78]. Gu et al. conducted a study evaluating the effect of FOS use on the composition of the gut microbiota in mice. It was observed that the relative abundance of *Actinobacteria* increased significantly, especially *Bifidobacterium* and *Coprococcus*, while *Bacteroidetes* and *Proteobacteria* decreased [79]. Another study carried out on mice models showed that FOS leads to a reduction of inflammatory parameters, such as IL-6 and TNF-α. A significantly higher concentration of SCFA in the serum and the feces was reported in the group using this prebiotic [80].

#### 4.2.2. Galactooligosaccharides

GOS are composed of galactopyranosyl molecules and are naturally found in lentils, chickpeas, and beans. This prebiotic is synthesized from soybeans and lactose (cow’s milk) [81]. The positive effect of GOS on the gut microbiota is especially visible in the group of newborns and infants. GOS (along with FOS) is an ingredient added to milk mixtures due to its beneficial effect on the number of lactic acid bacteria—*Lactobacillus* and *Bifidobacterium* [82,83,84]. However, a beneficial effect of GOS on these bacteria was demonstrated in all age groups. This probiotic contributed to the reduction of pathogenic *Clostridium*. The intake of the GOS mixture increased the concentration of IL-10 and IL-8 and decreased IL-1β [85]. The consumption of GOS in a group of elderly people for 10 weeks at a dose of 5.5 g/day caused a change in their intestinal microbiota composition. An increase in the number of *Lactobacillus-Enterococcus* spp., *Bifidobacterium* spp., and *C. cocoides-E* was noticed in the GOS group compared to the placebo. In addition, the number of *E. coli*, *Bacteroides* spp., *Desulfovibrio* spp., and the *C. histolyticum* group was decreased. It was observed that the intake of GOS caused an increase in NK cell activity and an increase in IL-10 production. In contrast, the concentration of IL-1β, IL-6, and TNF-α was decreased, indicating an anti-inflammatory effect of GOS [86]. The most beneficial are prebiotics containing both FOS and GOS due to their positive effect on the intestinal microbiota.

#### 4.2.3. Inulin

Inulin is a fructan, a polysaccharide composed of fructose molecules linked by a β-1,2-glycosidic bond. It occurs in artichoke, garlic, onion, shallot, leek, salsify, scorzonera, asparagus, chicory, and banana. Inulin is used as a sugar and fat replacer. It is an ingredient of functional food due to its positive effect on gastric health [87]. In a 2-week study, the effect of eating a diet rich in vegetables containing large amounts of inulin (average consumption of inulin 15 g/day) on the intestinal microbiome was assessed. A three-fold increase in the number of bacteria from the *Bifidobacterium* genus and a downward trend in the number of *Oxalobacteraceae* family were reported. After three weeks from the end of the intervention, the bacterial content in the feces returned to the initial values [88]. In other studies, an increase in the quantity of *Bifidobacterium* was also noticed after consuming inulin-rich foods. Ramnani et al. assessed the effect of consuming 5 g of inulin per day in shots with Jerusalem artichoke. *Bifidobacteria* levels were significantly higher in a group with inulin. They also noted an increase in the *Lactobacillus*/*Enterococcus* ratio [89]. Kleessen et al. carried out a randomized, double-blind, placebo-controlled study with snack bars that included chicory inulin (CH), Jerusalem artichoke (JA) inulin, and without inulin. The total number of bacteria after consuming the inulin bars and the placebo bars was the same. People consuming CH or JA had a lower ratio of *Bacteroides/Prevotella* than the placebo group. Potential pathogenic bacteria *Clostridium histolyticum* and *C. lituseburense* were less frequently isolated in the inulin group [90]. The consumption of 12 g/day of inulin derived from chicory for four weeks in healthy adults with constipation led to an increase in the amount of *Anaerostipes* spp. and *Bifidobacterium*. There was a decline in the population of *Bilophila*, which was associated with a reduction in the incidence of constipation [91]. A study on animal models showed that inulin consumption was associated with a reduction in the expression of genes encoding proinflammatory factors, such as IL-1β, IL-6, TLR4, a dendritic cell marker (CD11c), and Ikk kinase ε (IKKε) [92]. In a study by Li et al., 6-week intake of inulin in mice with type 2 diabetes decreased LPS, IL-6, and TNF-α levels. An increase in the concentration of anti-inflammatory IL-10 was observed. There was an increase in the relative abundance of *Cyanobacteria* and *Bacteroides* and a decrease in the abundance of *Ruminiclostridium*. *Cyanobacteria* and *Bacteroides* were positively correlated with IL-10. The amount of *Deferribacteres*, *Tenericutes*, *Mucispirillum*, and *Ruminiclostridium* bacteria was correlated with IL-6 and TNF-α [93].

#### 4.2.4. Resistant Starch

Resistant starch (RS) is composed of α-linked glucose molecules that are resistant to hydrolysis in the small intestine due to resistance to digestive amylases. There are several types of resistant starch and the best known are found in whole grains and legumes, starch with high amylose content, and retrograded starch (e.g., starch in cooked and then cooled potatoes) [94]. Guy et al. compared the effects of a diet high in non-starch polysaccharides (NSPs) with a diet high in NSPs and rich in resistant starch. In that study, 46 healthy people participated in a 14-week dietary intervention. A significant increase in the number of *Ruminococcus bromii* in the RS group was observed [95]. A placebo-controlled study assessed the effect of supplementation with resistant starch derived from potatoes on the composition of the gut microbiota in the elderly (over 70 years) and in people aged 30–50 years. After 12 weeks of prebiotic therapy, a significant increase in the number of *Bifidobacterium* was demonstrated in both age groups. In addition, an increase in the concentration of butyrate in the stool was noted in the group of elderly people compared to a placebo [96]. Studies conducted among patients with chronic kidney disease indicated a positive effect of the consumption of resistant starch on the composition of the intestinal microbiota. An increase in the amount of *Bacteroides*, *Bifidobacteria*, *Lactobacilli*, and *Ruminococcus bromii* was noticed [97,98]. Zhang et al. carried out a study on mice models. Mice were on a high-fat diet with RS supplementation. The results showed a reduction in the number of some bacteria (*Helicobacter*, *Ruminiclostridium 9*, *Tyzzerella*, *Oscillibacter*, *Coprococcus 1*, *Lachnoclostridium*, *Desulfovibrio*). In the RS group, a decrease in the parameter assessing the tightness of the intestinal barrier, LPS, in serum and feces was observed. Inflammation parameters were also decreased (decreased IL-2 expression in the colon and IL-4 and TNF-α in the liver). The intake of resistant starch increased the concentration of SCFA in the colon [99].

#### 4.2.5. CLA, PUFA

Conjugated linoleic acid and polyunsaturated fatty acids are also classified as probiotics. CLA is found in milk, dairy products, and meat and PUFAs in oil plants, oils, and fish. The effect of the intake of CLA on the gut microbiota of mice was assessed. Significant growth in *Bacteroides/Prevotella* and mucin-degrading *A*. *muciniphila* was demonstrated [100]. Supplementation with eicosapentaenoic acid (EPA), docosahexaenoic acid (DHA), and the consumption of vegetable oils or fish contributes to an increase in the numbers of *Bifidobacterium*, *Oscillospira*, and *Akkermansiaceae*. In some studies, there was a lower amount of *Enterobacteria,* and some pathogenic bacteria, such as *Escherichia*, *Streptococcus*, and *Clostridium*, were observed [101,102]. Younge et al. assessed the effect of enteral supplementation with fish oil and safflower oil on the composition of the intestinal microbiome in premature infants with an enterostomy. Greater bacterial diversity was reported after PUFA supplementation. At the same time, the levels of *Streptococcus*, *Clostridium*, and many pathogenic bacteria from the *Enterobacteriaceae* family decreased [103]. The consumption of n-3 PUFAs also influences the tightness of the intestinal barrier. It was noted that higher consumption of EPA and DHA by pregnant women was associated with lower serum zonulin concentration [104]. Consumption of n-3 PUFAs inhibits the production of proinflammatory cytokines induced by LPS and the NF-κB pathways. These PUFAs promote the release of anti-inflammatory agents, such as IL-10, and may reduce intestinal inflammation by promoting the induction of regulatory T cells (Tregs) and reducing interleukin 17 (IL-17) production [105,106]. Unbalanced consumption of n-3/n-6 PUFAs may lead to dysbiosis of the gut microbes, especially a significant increase in the *Firmicutes* to *Bacteroidetes* ratio (F/B ratio), leading to overweight and obesity [107]. A high intake of omega-6 fatty acids may increase the proportion of LPS-producing and proinflammatory bacteria [108].

#### 4.2.6. Polyphenols

**Figure 3:**
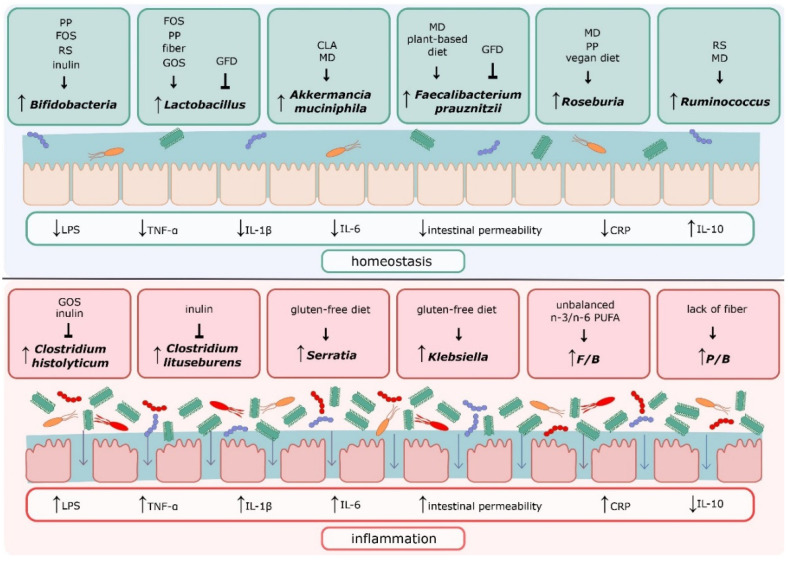
Effect of nutritional interventions on specific bacteria. Abbreviations: PP—polyphenols; FOS—fructooligosaccharides; RS—resistant starch; GOS—galactooligosaccharides; GFD—gluten-free diet; CLA—conjugated linoleic acid; MD—Mediterranean diet; LPS—lipopolysaccharide; TNF-α—tumor necrosis factor α; IL-1ß—interleukin 1ß; IL-6—interleukin 6; CRP—C-reactive protein; IL-10—interleukin 10.

The group of prebiotics also includes polyphenols, such as phenolic acids, flavonoids, stilbenes, and lignans. These compounds are found in vegetables, fruits, tea, coffee, and wine and have antioxidant, anti-inflammatory, and anticancer properties [109,110]. Research indicates that polyphenols also have a positive effect on the composition of the gut microbiota. They promote the growth of *Lactobacillus* and *Bifidobacteria* and inhibit the growth of potentially pathogenic bacteria, such as *Staphylococcus* sp. [111]. Moreno-Indias et al. conducted a 30-day study evaluating the effect of consuming red wine polyphenols on the microbiota of obese people with metabolic syndrome. Red wine polyphenols decreased the number of *Escherichia coli* and *Enterobacter cloacae* and increased fecal bifidobacteria, *Lactobacillus*, *Faecalibacterium prausnitzii*, and *Roseburia*. In addition, *Bifidobacterium* growth, caused by red wine intake, was associated with a reduction in plasma LPS levels [112]. The administration of polyphenol-rich oolong tea by mice for four weeks allowed an increase in the diversity of intestinal bacteria and a large increase in *Bacteroidetes* with a decrease in *Firmicutes* [113]. A schematic summary of the effect of nutritional interventions on selected bacteria and inflammation is presented in Figure 3.

### 4.3. Probiotics

Probiotics are living microorganisms that, when administered in defined amounts, provide health benefits to the host. They must be characterized at the genus, species, and strain level in the scientific nomenclature. In addition, the strains contained in the preparation must be registered in the international culture collection. The properties of the probiotics must be demonstrated in health benefits in at least one human trial and its safety must be proven in its intended use. It is also required that probiotics contain an adequate amount of living organisms for a beneficial effect on health throughout their shelf life [114].

Wang et al. proved that a 12-week supplementation with each of the probiotic strains of *Bifidobacterium animalis subsp. lactis I-2494* and *Lactobacillus paracasei CNCM I-4270* and *L. rhamnosus I-3690* in an animal model reduced the effects of a high-fat diet, i.e., reduced body weight gain, improved glucose metabolism, and reduced fatty liver. It also significantly reduced the infiltration of proinflammatory macrophages into adipose tissue, which is a subcutaneous cause of chronic adipose tissue inflammation and thus obesity-related complications. Additionally, *Bifidobacterium animalis subsp. lactis I-2494* significantly reduced TNF-α expression in the liver and adipose tissue as well as lowering the serum LBP concentration [115].

Just as in the animal model, in humans, probiotics can modulate chronic low-grade inflammation, particularly by inhibiting the NF-κB pathway and reducing cytokines [116]. Lactobacillus plantarum 299v administration led to an improvement in the inflammatory profile by decreasing IL-6 along with a reduction in risk factors of cardiovascular disease in smokers [117]. Fermented milk containing *Lactobacillus helveticus R389* reduced the secretion of IL-6 while inducing the secretion of interleukin 10, which has anti-inflammatory properties [118]. Supplementation with the *Akkermancia muciniphila*, a mucin-degrading bacterium, increased the thickness of the mucus and decreased the concentration of lipopolysaccharide in the serum. In the group with probiotics, fasting glucose concentration and glucose tolerance improved [119]. A meta-analysis describing 42 randomized placebo-controlled clinical trials showed that the intake of probiotics significantly reduces serum hs-CRP, TNF-α, IL-6, IL-12, and IL-4 with a simultaneous increase in the anti-inflammatory cytokine IL-10 [120]. Additionally, the benefits of taking probiotics have been noted in studies on various conditions, including metabolic syndrome, liver diseases, coronary heart disease, rheumatoid arthritis, and major depressive disorder, which are mentioned in Table 1.

## 5. Conclusions

An unhealthy diet, diseases, lack of exercise, sleep disorders, exposure to nicotine, drugs, and many other factors can lead to an imbalance in the gut microbiota [127]. Dysbiosis and increased intestinal permeability may contribute to inflammation and lead to metabolic disorders or exacerbate existing conditions. Inflammation can be caused by the presence of microorganisms and their structural elements and the products of their metabolism. Low-grade chronic inflammation and dysbiosis intensify each other, creating a vicious circle. Modifying the microbiota by changing the diet and using prebiotics and probiotics might restore microbiome balance and reduce inflammation along with improving metabolic status. However, the composition of the intestinal microbiota is unique for every human being. As a result, there is no universal method of microbiota modification that would bring health benefits. Attempts to modify the microbiota should be based on the type of dysbiosis [128]. However, the current state of knowledge on the subject is not sufficient and more research is required on many levels to understand how to modify the microbiota to improve the inflammatory profile.

## Figures and Tables

**Table 1:** Literature review on probiotics’ effects in various health conditions.

Reference	Health Condition	Sample Size	Probiotics	Duration	Effect in Inflammation	Other Effects
Bernini et al. 2015 [121]	metabolic syndrome	26 probiotic group 25 control group	fermented milk with 2.72 × 10^10^ CFU *Bifidobacterium lactis* HN019	45 days	↓ TNF-α ↓ IL-6	↓ BMI ↓ total cholesterol ↓ LDL
Akkasheh et al. 2016 [122]	major depressive disorder	20 probiotic group 20 control group	*Lactobacillus acidophilus* (2 × 10^9^ CFU/g), *Lactobacillus casei* (2 × 10^9^ CFU/g), *Bifidobacterium bifidum* (2 × 10^9^ CFU/g)	8 weeks	↓ hs-CRP	↓ BDI total scores ↓ insulin ↓ HOMA-IR ↑ glutathione
Zamani et al. 2016 [123]	rheumatoid arthritis	30 probiotic group 30 control group	*Lactobacillus acidophilus* (2 × 10^9^ [CFU]/g), *Lactobacillus casei* (2 × 10^9^ CFU/g), *Bifidobacterium bifidum* (2 × 10^9^ CFU/g)	8 weeks	↓ hs-CRP	↑ DAS28 ↓ insulin ↓ HOMA-B ↓ total cholesterol ↓ LDL
Moludi et al. 2021 [124]	coronary artery disease	22 probiotic group + caloric restriction 22 control group + caloric restriction	*Lactobacillus rhamnosus* GG (1.6 × 10^9^ CFU)	12 weeks	↓ IL-1ß	↓ LPS
Han et al. 2015 [125]	alcoholic hepatitis	60 probiotic group + alcohol abstinence 57 control group + alcohol abstinence	*Lactobacillus subtilis/Streptococcus faecium* (1500 mg/day)	7 days	↓ TNF-α	↓ LPS
Kobyliak et al. 2018 [126]	Non-alcoholic fatty liver disease	30 probiotic group 28 control group	*Lactobacillus + Lactococcus* (6 × 10^10^ CFU/g), *Bifidobacterium* (1 × 10^10^ CFU/g), *Propionibacterium* (3 × 10^10^ CFU/g), *Acetobacter* (1 × 10^6^ CFU/g)	8 weeks	↓ TNF-α ↓ IL-6	↓ liver fat ↓ AST ↓ GGT

Aberrations: UCF—colony-forming unit; TNF-α—tumor necrosis factor α; IL-6—interleukin 6; BMI—body mass index; LDL—low-density lipoprotein; hs-CRP—high-sensitivity C-reactive protein; BDI—Beck Depression Inventory; HOMA-IR—homeostasis model assessment of insulin resistance; DAS—Disease Activity Score of 28 joints; HOMA-B—homeostatic model assessment-B cell function; IL-1ß—interleukin 1ß; LPS—lipopolysaccharide; AST—aspartate aminotransferase; GGT—gamma-glutamyl transferase; ↑—significantly increased; ↓—significantly decreased.

## Data Availability

Not applicable.
